# In Situ Femtosecond-Laser-Induced Fluorophores on Surface of Polyvinyl Alcohol for H_2_O/Co^2+^ Sensing and Data Security

**DOI:** 10.3390/s21227755

**Published:** 2021-11-22

**Authors:** Weiliang Chen, Jichao Gao, Jie Tian, Jingyu Zhang

**Affiliations:** Wuhan National Laboratory for Optoelectronics, Huazhong University of Science and Technology, Wuhan 430074, China; chenweilliang@163.com (W.C.); xswb1gjc@163.com (J.G.); jietian0016@163.com (J.T.)

**Keywords:** data security, fluorescence, laser ablation, materials processing, microfluidics

## Abstract

In situ fluorophores were induced on polyvinyl alcohol (PVA) bulk materials by direct femtosecond laser writing. The generation of fluorophores was ascribed to localized laser-assisted carbonization. The carbonization of PVA polymers was confirmed through X-ray photoelectron spectroscopy analysis. The distinct fluorescence responses of fluorophores in various solutions were harnessed for implementing in situ reagent sensors, metal ion sensors, data encryption, and data security applications. The demonstrated water detection sensor in acetone exhibited a sensitivity of 3%. Meanwhile, a data encryption scheme and a “burn after reading” technique were demonstrated by taking advantage of the respective reversible and irreversible switching properties of the in situ laser-induced fluorophores. Taking a step further, a quantitative cobalt ion measurement was demonstrated based on the concentration-dependent fluorescence recovery. Combined with a laser-induced hydrophilic modification, our scheme could enable “lab-on-a-chip” microfluidics sensors with arbitrary shape, varied flow delay, designed reaction zones, and targeted functionalities in the future.

## 1. Introduction

Fluorescent materials have extensive applications in biological imaging [1], drug delivery [2], sensors [3,4,5,6], data storage [7], and anti-counterfeiting encryption [8,9]. Massive production methods, including hydrothermal, solvothermal, and pyrolysis methods, have been widely used for preparing fluorescent materials. However, these methods have traditionally faced challenges in the fabrication of microscale arbitrary fluorescence-based functional devices.

Owing to the ease of creating highly-localized and targeted material modifications by femtosecond direct laser writing, microscale functional devices can be fabricated in situ [10,11]. In addition, in situ femtosecond-laser-induced fluorophores in transparent organic materials have enabled a growing number of applications, such as optical data storage [12,13], microfluidics [14], and patterning [15]. The stimuli-responsive properties of fluorescent materials have also been implemented for information security encryption [16,17].

In this work, fluorophores were generated in situ by femtosecond-laser-induced direct writing on the surface of polyvinyl alcohol (PVA). This property was employed for multi-level optical data storage. Excited fluorescence was quenched by organic solvents with a hydroxyl group. A lab-on-a-chip water sensor was fabricated by harnessing this response behavior. In addition, a reversible data encryption-decryption scheme based on water-responsive fluorophores was demonstrated. Taking a step further, we present a “burn after reading” data security technique based on metal-ion-induced permanent quenching. By grading the recovered fluorescence intensity, quantitative cobalt ion measurement was implemented.

## 2. Materials and Methods

PVA (1799) powder was purchased from Aladdin. PVA (1 g) was dissolved in deionized water (10 mL) by continuous stirring. Then, the solvent was maintained at 90 °C until complete dissolution. To prepare the PVA film, the PVA solution was cast on a glass slide and dried at room temperature. Aqueous solutions of Co^2+^ were obtained by dissolving cobalt chloride in deionized water.

**Figure 1:**
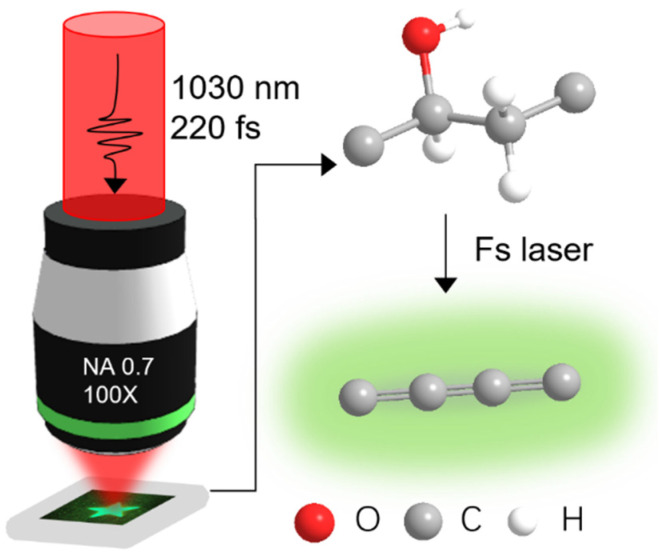
Schematic of a pattern written in PVA using a femtosecond laser (**left**). Schematic of the carbonization and dehydration processes after femtosecond-laser-based irradiation (**right**).

Patterns were written on the surface of the PVA samples by direct laser writing (Figure 1). A femtosecond laser system (Light Conversion, Pharos, Vilnius, Lithuania) with a central wavelength of 1030 nm, repetition rate of 100 kHz, average power of 30 mW, and pulse width of 220 fs was employed. The pulse energy was manipulated using a programmed rotating half-wave plate and a linear polarizer. A dry objective lens with a numerical aperture of 0.7 was used for focusing the incident beam. An XYZ translation stage (Aerotech, Pittsburgh, PA, USA) was used for positioning and scanning the samples. An excitation laser at 488 nm with a power of 2.5 mW was used in all of the fluorescence imaging experiments in this work.

## 3. Results and Discussion

**Figure 2:**
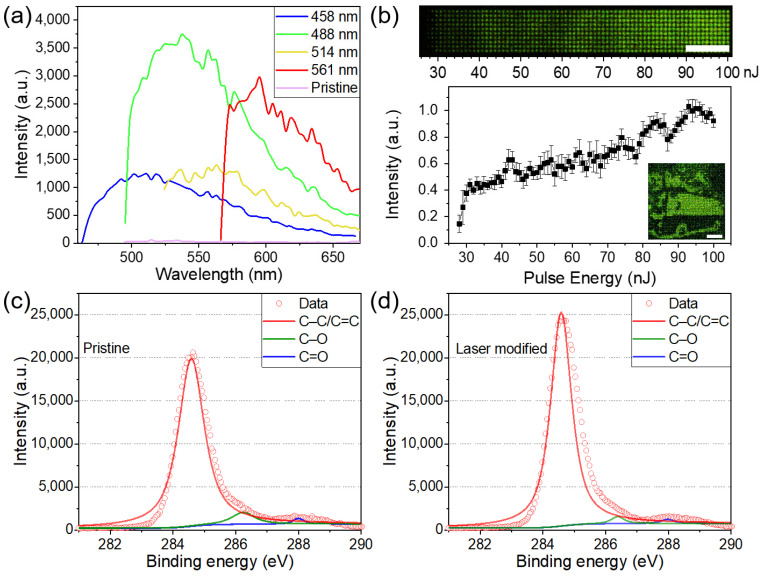
(**a**) Photoluminescence spectra of modified PVA samples for different excitation wavelengths and pristine PVA sample for the excitation wavelength of 488 nm. (**b**) Confocal fluorescence microscopy image of voxel array excited at 488 nm. Fluorescence intensity as a function of pulse energy. The inset is a 4-level picture of a bus. The scale bars are 50 μm. XPS spectra of (**c**) the pristine PVA sample and (**d**) the laser-modified PVA sample.

The fluorescence emission spectra of the PVA samples were obtained using a Zeiss LSM 710 confocal microscope. For the fluorescence measurement, a 1 × 1 mm area was fabricated on the surface with a line scanning speed of 1 mm/s and line separation of 10 µm. The untreated PVA sample had a negligible fluorescence signal, whereas the modified samples exhibited a fluorescence intensity that was two orders of magnitude higher than that of the pristine sample (Figure 2a). The fluorescence emission spectrum displayed a typical excitation-dependent feature. The emission peaks gradually shifted to longer wavelengths with increasing excitation wavelength. A similar excitation-dependent emission characteristic was observed in carbon dots [18,19,20,21] and was explained by size heterogeneity [22] and variations in the surface group composition [23]. The fluorescence property can be employed for multi-level optical data storage (Figure 2b). As a proof-of-concept, a bus photo, composed of 50 × 50 4-level pixels, was written on PVA by single-pulse femtosecond laser irradiation (Figure 2b, inset).

The chemical changes in the PVA samples were characterized using X-ray photoelectron spectroscopy (XPS). The XPS data were recorded using an AXIS-ULTRA DLD-600W spectrometer. A 1 × 1 cm area was laser-processed on the surface for XPS measurements. The XPS tests were conducted on the regions with and without femtosecond laser irradiation (Figure 2c,d). In both cases, the XPS spectra were decomposed into the contributions of C–C/C=C, C–O, and C=O bonds, which were located at 284.6, 286.4, and 288.0 eV, individually. The fitting peak area results indicate that after laser irradiation the relative content of the C–C/C=C bonds increased by 13%. Such changes in chemical bonds signify the carbonization of PVA [24,25,26]. Meanwhile, dehydration of the PVA chain is expected to occur owing to laser-induced thermal effects. Fluorophore generation can be attributed to the introduction of nanoclusters [18], carbon quantum dots [19], and aromatic compounds [13,20]. However, the existence form of fluorophores remains to be investigated. Transmission electron microscopy (TEM) characterization of in situ generated fluorophores remains challenging [26].

**Figure 3:**
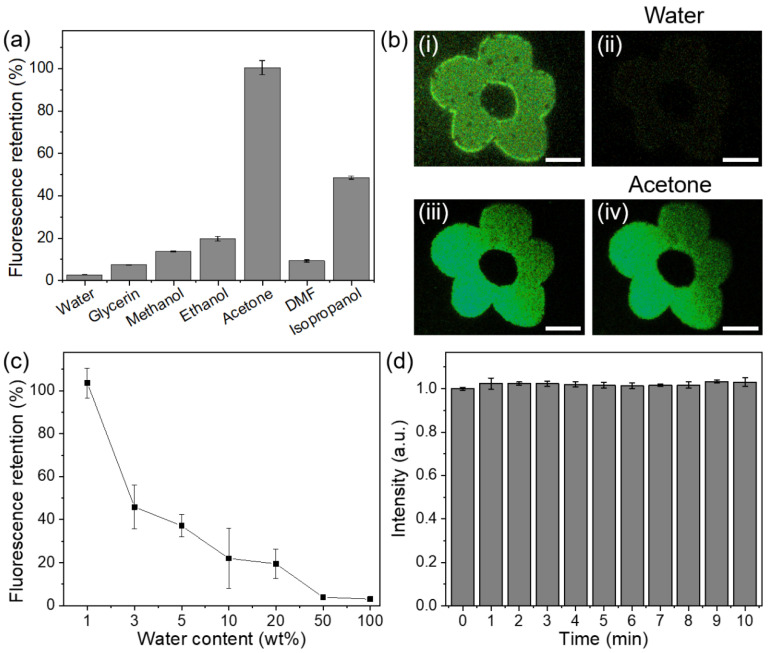
Reagent sensing characteristics of the fluorophores. (**a**) Fluorescence retention of different solvents on the laser-modified PVA samples. (**b**) Fluorescence images of two patterned PVA samples before and after water (**i**,**ii**) and acetone (**iii**,**iv**) dropping, respectively. The scale bars are 50 µm. (**c**) The fluorescence retention as a function of water content in acetone. (**d**) Fluorescence intensities of the laser-modified PVA after continuous exposure to 488-nm-wavelength excitation. The intensities are normalized to the first measurement.

As a reagent detection method, fluorophore-based sensors have the advantages of sensitivity and rapidity [27,28,29]. Combined with the in situ fabrication property of femtosecond-laser-based direct writing, “lab-on-a-chip” reagent sensors could be implemented. Following this idea, we tested the fluorescence responses of the modified PVA samples with different solvents. Eight identical micrometer-scale patterns were fabricated. Following the deposition of different solvents (water, glycerin, methanol, ethanol, acetone, DMF, isopropanol) on these patterns, the fluorescence response varied significantly. Dramatic fluorescence intensity reduction was observed, except in the case of acetone (Figure 3a). This phenomenon can be ascribed to the influence of intermolecular hydrogen bonds [30] and solvent polarity [31]. Water, glycerin, methanol, ethanol, and isopropanol have hydroxyl groups that may introduce hydrogen bonds to fluorophores, reducing the fluorescence intensity [32]. Although both dimethylformamide (DMF) and acetone are solvents without hydroxyl groups, their corresponding fluorescence responses are distinct. The retention discrepancy could be attributed to the relative difference in polarities [29]. Fluorescence images of the induced pattern response to water and acetone are shown in Figure 3b. A significant reduction in the fluorescence intensity after water addition is visible (2.6% remained). In contrast, a negligible intensity change was observed when the added solvent was acetone (98.2% remained). Thus, fluorescent sensors for detecting water in acetone can be fabricated. The intensities of the fluorescence response to acetone solutions with different water contents were measured (Figure 3c). The fluorescence retention decreased linearly with increasing water content. Even for an acetone solution with 3% water content, the fluorescence response was still significant (quenching more than 50%). Therefore, our method that uses laser-induced in situ fluorophores can enable simple and rapid water detection, for which only microliter-scale solvents are required. It is a promising method for sensing water in organic solvents that possess low polarity or are free of hydroxyl groups, including pentane, hexane, petroleum ether, cyclohexane, and isooctane.

To rule out the possible influence of laser-induced photobleaching that is common in confocal microscopy, we measured the fluorescence intensity of the structures during 10-min-long continuous laser irradiation with a fixed one-minute measurement interval (Figure 3d). No obvious decay of fluorescence intensity was observed.

**Figure 4:**
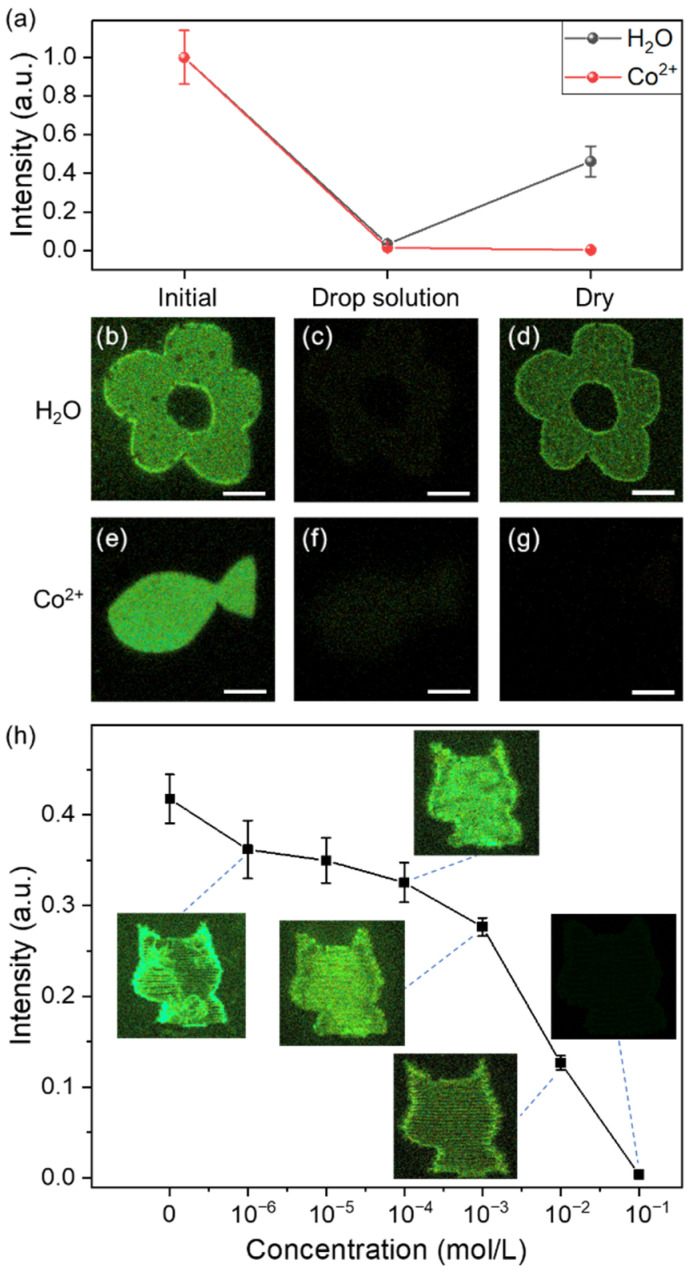
(**a**) Normalized fluorescence intensities of the laser-induced patterns following solvent deposition and drying. (**b**) A flower pattern fabricated using a femtosecond laser on the PVA surface, followed by 10 μL water dropping (**c**), and consequent heating at 35 °C for 2 h (**d**). (**e**) A fish pattern fabricated using a femtosecond laser on the PVA surface, followed by 10 μL Co^2+^ aqueous solution (0.1 mol/L) dropping (**f**), and consequent heating at 35 °C for 2 h (**g**). (**h**) Recovered fluorescence intensity as a function of cobalt ion concentration. The insets are corresponding patterns. The intensities are normalized to the measurement of the untreated laser-patterned sample. The scale bars are 50 µm.

It was also corroborated that fluorophores could respond to metal ions in the form of fluorescence property changes, as reported previously [33,34,35,36,37]. Assuming this characteristic, laser-induced in situ fluorophores could be used for implementing “burn after reading”, for data security. In Figure 4a, the fluorophores were permanently turned off after dropping the Co^2+^ aqueous solution (0.1 mol/L). The fluorescence intensity remained negligible after drying (from 1.9% to 0.1%). The irreversible quenching is attributed to the reaction in which the empty orbital of Co^2+^ is coordinated with the electrons of carbonized clusters so that the fluorescence is still inhibited after drying [34,35,36]. In addition to Co^2+^, other transition metal ions with empty orbitals, such as Cu^2+^, Ni^2+^, Fe^3+^, Cd^2+^, Pb^2+^, and Hg^2+^, may also be exploited as quenching agents. An example is presented in Figure 4e–g. In addition to data security applications, laser-induced fluorophores could be used for quantitative detection of metal ions, utilizing the quenching property. A series of laser-patterned PVA samples was dripped with Co^2+^ aqueous solutions of different concentrations, respectively (0 to 10^−1^ mol/L). After subsequent drying, the fluorescence recovered partially depending on the cobalt ion concentration (Figure 4h). Based on this principle, quantitative measurement of metal ion concentrations in solution can be implemented.

**Figure 5:**
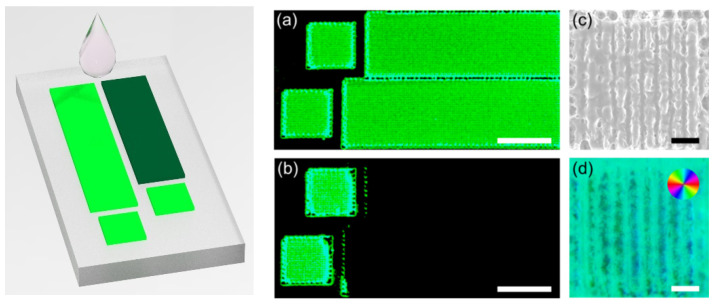
Hydrophilic surface laser patterning. Fluorescence images of the hydrophilic patterns (**a**) before and (**b**) after dropping the Co^2+^ aqueous solution (0.1 mol/L) at the far right of the patterns. The gaps between the left squares and the right rectangles are 100 μm. The scale bars are 500 μm. (**c**) Corresponding SEM and (**d**) birefringent slow axis orientation images of the laser-patterned PVA surface. Pseudo colors (inset (**d**)) indicate the direction of the slow axis. The scale bars are 10 μm. Laser parameters: 300 mW, 10 mm/s scanning speed, 1 MHz repetition rate, 0.25 NA objective lens.

Besides inducing fluorophores on the surface of PVA, another advantage of femtosecond laser processing is to create functionalized surface structures [38]. In our experiment, the laser-modified PVA surface was shown to possess hydrophilic property (Figure 5a,b). The Co^2+^ aqueous solution inhibited the fluorescence signal in the targeted rectangular regions, whereas the fluorescence in the square regions was almost unaffected. The fluid was mainly localized in the targeted area and could not across the 100 μm gap. Intriguingly, the porous hydrophilic modification (Figure 5c) exhibited form birefringence property (Figure 5d), owing to the laser-induced periodic surface structures [39]. The average retardance value was 18.5 ± 1.0 nm (Figure 5d). We envision that drainage channels with different fluid flow delays and designed reaction zones could be fabricated by tuning the laser parameters such as scanning direction, velocity, light intensity, and polarization. In addition, surface multi-functional devices could be readily fabricated in the near future combining the stimuli-responsive properties of the laser-induced fluorophores and femtosecond laser micro/nano-machining.

## 4. Conclusions

In conclusion, in situ surface fluorophores were obtained by femtosecond-laser-based processing of transparent organic material PVA. The obtained fluorophores were characterized in terms of their fluorescence emission spectra and XPS. The carbonization of PVA in the laser-modified region was confirmed. Such change in the material was due to the laser-induced plasma ablation and the subsequent thermal effects. To demonstrate whether the induced fluorophores could be used for optical data storage, a four-level image was printed on PVA. Owing to the fluorescence response to water, fluorophores were implemented for fabricating a water sensor. In addition, the off-on switching and metal-ion permanent quenching properties of these fluorophores were demonstrated for data security and metal ion measurement applications. Combined with the laser-induced hydrophilic modification, such femtosecond laser-induced in situ fluorophores are promising for various applications, such as “lab-on-a-chip” detection, fluorescent patterning, data storage, and data security. The organic substrate, as a platform of diverse composition, is desired for the polymorphism of femtosecond-enabled in situ fabrications. Our work is likely to shed some light on the future development of in situ femtosecond-laser-printed functional fluorescent devices based on multi-component organic/inorganic materials. Using these materials as a carbon source, we expect that the properties of the femtosecond-laser-induced carbon fluorophores can be selectively tuned by changing the chemical precursors, such as nitrogen-containing, boron-containing, and sulfur-containing materials.

## Data Availability

The data presented in this study are available on request from the corresponding author.
